# Approximate maximum likelihood estimation for stochastic chemical kinetics

**DOI:** 10.1186/1687-4153-2012-9

**Published:** 2012-07-18

**Authors:** Aleksandr Andreychenko, Linar Mikeev, David Spieler, Verena Wolf

**Affiliations:** 1 Computer Science Department, Saarland University, 66123 Saarbrücken, Germany

## Abstract

Recent experimental imaging techniques are able to tag and count molecular populations in a living cell. From these data mathematical models are inferred and calibrated. If small populations are present, discrete-state stochastic models are widely-used to describe the discreteness and randomness of molecular interactions. Based on time-series data of the molecular populations, the corresponding stochastic reaction rate constants can be estimated. This procedure is computationally very challenging, since the underlying stochastic process has to be solved for different parameters in order to obtain optimal estimates. Here, we focus on the maximum likelihood method and estimate rate constants, initial populations and parameters representing measurement errors.

## Introduction

During the last decade stochastic models of networks of chemical reactions have become very popular. The reason is that the assumption that chemical concentrations change deterministically and continuously in time is not always appropriate for cellular processes. In particular, if certain substances in the cell are present in small concentrations the resulting stochastic effects cannot be adequately described by deterministic models. In that case, discrete-state stochastic models are advantageous because they take into account the discrete random nature of chemical reactions. The theory of stochastic chemical kinetics provides a rigorously justified framework for the description of chemical reactions where the effects of molecular noise are taken into account [1]. It is based on discrete-state Markov processes that explicitly represent the reactions as state-transitions between population vectors. When the molecule numbers are large, the solution of the deterministic description of a reaction network and the mean of the corresponding stochastic model agree up to a small approximation error. If, however, species with small populations are involved, then only a stochastic description can provide probabilities of events of interest such as probabilities of switching between different expression states in gene regulatory networks or the distribution of gene expression products. Moreover, even the mean behavior of the stochastic model can largely deviate from the behavior of the deterministic model [2]. In such cases the parameters of the stochastic model rather then the parameters of the deterministic model have to be estimated [3-5].

Here, we consider noisy time series measurements of the system state as they are available from wet-lab experiments. Recent experimental imaging techniques such as high-resolution fluorescence microscopy can measure small molecule counts with measurement errors of less than one molecule [6]. We assume that the structure of the underlying reaction network is known but the stochastic reaction rate constants of the network are unknown parameters. Then we identify rate constants that maximize the likelihood of the time series data. Maximum likelihood estimators are the most popular estimators since they have desirable mathematical properties. Specifically, they become minimum variance unbiased estimators and are asymptotically normal as the sample size increases.

Our main contribution consists in devising an efficient algorithm for the numerical approximation of the likelihood and its derivatives w.r.t. the stochastic reaction rate constants. Furthermore, we show how similar techniques can be used to estimate the initial molecule numbers of a network as well as parameters related to the measurement error. We also present extensive experimental results that give insights about the identifiability of certain parameters. In particular, we consider a simple gene expression model and the identifiability of reaction rate constants w.r.t. varying observation interval lengths and varying numbers of time series. Moreover, for this system we investigate the identifiability of reaction rate constants if the state of the gene cannot be observed but only the number of mRNA molecules. For a more complex gene regulatory network, we present parameter estimation results where different combinations of proteins are observed. In this way we reason about the sensitivity of the estimation of certain parameters w.r.t. the protein types that are observed.

Previous parameter estimation techniques for stochastic models are based on Monte-Carlo sampling [3,5] because the discrete state space of the underlying model is typically infinite in several dimensions and a priori a reasonable truncation of the state space is not available. Other approaches are based on Bayesian inference which can be applied both to deterministic and stochastic models [7-9]. In particular, approximate Bayesian inference can serve as a way to distinguish among a set of competing models [10]. Moreover, in the context of Bayesian inference linear noise approximations have been used to overcome the problem of large discrete state spaces [11].

Our method is not based on sampling but directly calculates the likelihood using a dynamic truncation of the state space. More precisely, we first show that the computation of the likelihood is equivalent to the evaluation of a product of vectors and matrices. This product includes the transition probability matrix of the associated continuous-time Markov process, i.e., the solution of the Kolmogorov differential equations (KDEs), which can be seen as a matrix-version of the chemical master equation (CME). Solving the KDEs is infeasible because of the state space of the underlying Markov model is very large or even infinite. Therefore we propose an iterative approximation algorithm during which the state space is truncated in an on-the-fly fashion, that is, during a certain time interval we consider only those states that significantly contribute to the likelihood. This technique is based on ideas presented in [12], but here we additionally explain how the initial molecule numbers can be estimated and how an approximation of the standard deviation of the estimated parameters can be derived. Moreover, we provide more complex case studies and run extensive numerical experiments to assess the identifiability of certain parameters. In these experiments we assume that not all molecular populations can be observed and estimate parameters for different observation scenarios, i.e., we assume different numbers of observed cells and different observation interval lengths. We remark that this article is an extension of a previously published extended abstract [13].

The article is further organized as follows: After introducing the stochastic model in Section“Discrete-state stochastic model”, we discuss the maximum likelihood method in Section “Parameter inference” and present our approximation method in Section “Numerical approximation algorithm”. Finally, we report on experimental results for two reaction networks in Section “Numerical results”.

## Discrete-state stochastic model

According to Gillespie’s theory of stochastic chemical kinetics, a well-stirred mixture of *n* molecular species in a volume with fixed size and fixed temperature can be represented as a continuous-time Markov chain {**X**(*t*),*t* ≥ 0} [1]. The random vector **X**(*t*)=(*X*_1_(*t*),…,*X*_*n*_(*t*)) describes the chemical populations at time *t*, i.e., *X*_*i*_(*t*) is the number of molecules of type *i* ∈ {1,…,*n*} at time *t*. Thus, the state space of **X **is $Z_{+}^{n}={\left\{0,1,\ldots \right\}}^{n}$. The state changes of **X**are triggered by the occurrences of chemical reactions, which are of *m* different types. For *j* ∈ {1,…,*m*} let $v_{j}\in Z^{n}$ be the nonzero *change vector* of the *j*-th reaction type. Thus, if **X**(*t*)=**x** and the *j*-th reaction is possible in **x**, then **X**(*t* + *dt*)=**x** + **v**_*j*_ is the state of the system after the occurrence of the *j*-th reaction within the infinitesimal time interval *tt* + *dt*).

Each reaction type has an associated *propensity function*, denoted by *α*_1_,…,*α*_*m*_, which is such that *α*_*j*_(**x**)·*dt* is the probability that, given **X**(*t*)=**x**, one instance of the *j*-th reaction occurs within [*t*,*t* + *dt*). The value *α*_*j*_(**x**) is proportional to the number of distinct reactant combinations in state **x** and to the reaction rate constant *c*_*j*_. The probability that a randomly selected pair of reactants collides and undergoes the *j*-th chemical reaction within [*t*,*t* + *dt*) is then given by *c*_*j*_*dt*. The value *c*_*j*_depends on the volume and the temperature of the system as well as on the microphysical properties of the reactant species.

### Example 1

We consider the simple gene expression model described in [4] that involves three chemical species, namely DNA_ON_, DNA_OFF_, and mRNA, which are represented by the random variables *X*_1_(*t*), *X*_2_(*t*), and *X*_3_(*t*), respectively. The three possible reactions are DNA_ON_→DNA_OFF_, DNA_OFF_→DNA_ON_, and DNA_ON_→DNA_ON_+ mRNA. Thus, **v**_1_=(−1,1,0), **v**_2_=(1,−1,0), **v**_3_=(0,0,1). For a state **x**=(*x*_1_*x*_2_*x*_3_), the propensity functions are *α*_1_(**x**)=*c*_1_·*x*_1_, *α*_2_(**x**)=*c*_2_·*x*_2_, and *α*_3_(**x**)=*c*_3_·*x*_1_. Note that given the initial state **x**=(1,0,0), at any time, either the DNA is active or not, i.e. *x*_1_=0 and *x*_2_=1, or *x*_1_=1 and *x*_2_=0. Moreover, the state space of the model is infinite in the third dimension. For a fixed time instant *t* > 0, no upper bound on the number of mRNA is known a priori. All states **x**with $x_{3}\in Z_{+}$ have positive probability if *t* > 0 but these probabilities will tend to zero as *x*_3_→*∞*.

### The CME

For a state $x\in Z_{+}^{n}$ and *t* ≥ 0, let *p*(**x**,*t*) denote the probability Pr(**X**(*t*)=**x**), i.e., the probability that the process is in state **x** at time *t*. Furthermore, let **p**(*t*) be the row vector with entries *p*(**x**,*t*) where we assume a fixed enumeration of all possible states.

Given **v**_1_,…,**v**_*m*_, *α*_1_,…,*α*_*m*_, and some initial populations **x**(0)=(*x*_1_(0),…,*x*_*n*_(0))with *P*(**X**(0)=**x**(0))=1, the Markov chain **X**is uniquely specified and its evolution is given by the CME

(1) $$\frac{d}{\mathrm{dt}}p(t)=p(t)Q,$$

where *Q* is the infinitesimal generator matrix of **X**with *Q*(**xy**)=*α*_*j*_(**x**) if **y**=**x** + **v**_*j*_ and reaction type *j* is possible in state **x**. Note that, in order to simplify our presentation, we assume here that all vectors **v**_*j*_ are distinct. All remaining entries of *Q* are zero except for the diagonal entries which are equal to the negative row sum. The ordinary first-order differential equation in (1) is a direct consequence of the Kolmogorov forward equation but standard numerical solution techniques for systems of first-order linear equations cannot be applied to solve (1) because the number of nonzero entries in *Q* typically exceeds the available memory capacity for systems of realistic size. If the expected populations of all species remain small (at most a few hundreds) then the CME can be efficiently approximated using projection methods [14-16] or fast uniformization methods [17,18]. The idea of these methods is to avoid an exhaustive state space exploration and, depending on a certain time interval, restrict the analysis of the system to a subset of states.

We are interested in the partial derivatives of **p**(*t*) w.r.t. a certain parameter *λ* such as reaction rate constants *c*_*j*_, *j* ∈ {1,…,*m*} or initial populations *x*_*i*_(0), *i*∈{1,…,*n*}. Later, they will be used to maximize the likelihood of observations and to find optimal parameters. In order to explicitly indicate the dependence of **p**(*t*) on *λ* we may write **p**_*λ*_(*t*) instead of **p**(*t*) and *p*_*λ*_(**x**,*t*) instead of *p*(**x**,*t*). We define the row vector **s**_*λ*_(*t*) as the derivative of **p**_*λ*_(*t*) w.r.t. *λ*, i.e.,

$$s_{\lambda }(t)=\frac{\partial p_{\lambda }(t)}{\partial \lambda }=\underset{\Delta \to 0}{\lim}\frac{p_{\lambda +\Delta }(t)-p_{\lambda }(t)}{\Delta }.$$

We denote the entry in **s**_*λ*_(*t*) that corresponds to state **x** by *s*_*λ*_(**x**,*t*). Note that we use bold face for vectors. By (1), we find that **s**_*λ*_(*t*) is the solution of the system of ODEs

(2) $$\frac{d}{\mathrm{dt}}s_{\lambda }(t)=s_{\lambda }(t)Q+p_{\lambda }(t)\frac{\partial }{\partial \lambda }Q,$$

when choosing *λ*=*c*_*j*_ for *j* ∈ {1,…,*m*}. In this case, the initial condition is *s*_*λ*_(**x**,0)=0 for all **x** since *p*(**x**,0) is independent of *c*_*j*_. If the unknown parameter is the *i*-th initial population, i.e., *λ*=*x*_*i*_(0), then we get

(3) $$\frac{d}{\mathrm{dt}}s_{\lambda }(t)=s_{\lambda }(t)Q,$$

with initial condition $s_{\lambda }(0)=\frac{\partial }{\partial \lambda }p_{\lambda }(0)$ since *Q* is independent of *x*_*i*_(0). Similar ODEs can be derived for higher order derivatives of the CME.

## Parameter inference

Following the notation in [4], we assume that observations of the reaction network are made at time instances $t_{1},\ldots ,t_{R}\in R_{\geq 0}$ where *t*_1_ < ⋯ <*t*_*R*_. Since it is unrealistic to assume that all species can be observed, we assume w.l.o.g. that the species are ordered such that we have observations of *X*_1_,…,*X*_*d*_ for some fixed *d* with 1 ≤ *d* ≤ *n*, i.e. *O*_*i*_(*t*_*ℓ*_) is the observed number of species *i* at time *t*_*ℓ*_for *i* ∈ {1,…,*d*} and *ℓ* ∈ {1,…,*R*}. Let **O**(*t*_*ℓ*_)=(*O*_1_(*t*_*ℓ*_),…,*O*_*d*_(*t*_*ℓ*_))be the corresponding vector of observations. Since these observations are typically subject to measurement errors, we assume that *O*_*i*_(*t*_*ℓ*_)=*X*_*i*_(*t*_*ℓ*_) + *ε*_*i*_(*t*_*ℓ*_) where the error terms *ε*_*i*_(*t*_*ℓ*_) are independent and identically normally distributed with mean zero and standard deviation *σ*. Note that *X*_*i*_(*t*_*ℓ*_) is the true population of the *i*-th species at time *t*_*ℓ*_. Clearly, this implies that, conditional on *X*_*i*_(*t*_*ℓ*_), the random variable *O*_*i*_(*t*_*ℓ*_) is independent of all other observations as well as independent of the history of **X** before time *t*_*ℓ*_.

We assume further that we do not know the values of the rate constants **c**=(*c*_1_,…,*c*_*m*_) and our aim is to estimate these constants. Similarly, the initial populations **x**(0) and the exact standard deviation *σ*of the error terms are unknown and must be estimated. We remark that it is straightforward to extend the estimation framework such that a covariance matrix for a multivariate normal distribution of the error terms is estimated. In this way, different measurement errors of the species can be taken into account as well as dependencies between error terms.

Let *f * denote the joint density of **O**(*t*_1_),…,**O**(*t*_*R*_) and, by convenient abuse of notation, for a vector **x**_*ℓ*_=(*x*_1_,…,*x*_*d*_) let **X**(*t*_*ℓ*_)=**x**_*ℓ*_ represent the event that *X*_*i*_(*t*_*ℓ*_)=*x*_*i*_ for 1 ≤ *i* ≤ *d*. In other words, **X**(*t*_*ℓ*_)=**x**_*ℓ*_ means that the populations of the observed species at time *t*_*ℓ*_equal the populations of vector **x**_*ℓ*_. Note that this event corresponds to a set of states of the Markov process since *d* may be smaller than *n*. More precisely, $\mathrm{Pr}\left(X(t_{\ell })=x_{\ell }\right)=\underset{y:y_{i}=x_{i},i\leq d}{\sum }p(y,t_{\ell })$. Now the likelihood of the observation sequence **O**(*t*_1_),…,**O**(*t*_*R*_) is given by

(4) $$\begin{array}{l} ℒ=f\left(O(t_{1}),\ldots ,O(t_{R})\right)\\ \;=\underset{x_{1}}{\sum }\ldots \underset{x_{R}}{\sum }f\left(O(t_{1}),\ldots ,O(t_{R})\right)|\\ \;\;\left(\;\;\;\;\;X(t_{1})=x_{1},\ldots ,X(t_{R})=x_{R}\right)\\ \;\;\mathrm{Pr}\;\left(X(t_{1})=x_{1},\ldots ,X(t_{R})=x_{R}\right). \end{array}$$

Note that  ℒ depends on the chosen rate parameters **c** and the initial populations **x**(0) since the probability measure Pr(·) does. Furthermore,  ℒ depends on *σ*since the density *f * does. When necessary, we will make this dependence explicit by writing ℒ(x(0),c,σ) instead of  ℒ. We now seek constants **c**^∗^, initial populations **x**(0) and a standard deviation *σ*^∗^such that

(5) $$ℒ(x{\left(0\right)}^{*},c^{*},\sigma^{*})=\underset{x(0),\sigma ,c}{\max}ℒ(x(0),c,\sigma )$$

where the maximum is taken over all *σ* > 0 and vectors **x**(0), **c** with all components strictly positive. This optimization problem is known as the maximum likelihood problem [19]. Note that **x**(0)^∗^, **c**^∗^ and *σ*^∗^are random variables because they depend on the (random) observations **O**(*t*_1_),…,**O**(*t*_*R*_).

If more than one sequence of observations is made, then the corresponding likelihood is the product of the likelihoods of all individual sequences. More precisely, if **O**^*k*^(*t*_*l*_) is the *k*-th observation that has been observed at time instant *t*_*l*_where *k* ∈ {1,…,*K*}, then we define ${ℒ}_{k}(x(0),c,\sigma )$ as the probability to observe **O**^*k*^(*t*_1_),…,**O**^*k*^(*t*_*R*_) and maximize

(6) $$\overset{K}{\underset{k=1}{\prod }}{ℒ}_{k}(x(0),c,\sigma ).$$

In what follows, we concentrate on expressions for ${ℒ}_{k}(x(0),c,\sigma )$ and $\frac{\partial }{\partial c_{j}}{ℒ}_{k}(x(0),c,\sigma )$. We first assume *K*=1 and drop index *k*. We consider the case *K* > 1 later. In (4) we sum over all population vectors **x**_1_,…,**x**_*R*_of dimension *d* such that Pr(**X**(*t*_*ℓ*_)=**x**_*ℓ*_,1 ≤ *ℓ* ≤ *R*) > 0. Since **X** has a large or even infinite state space, it is computationally infeasible to explore all possible sequences. In Section “Numerical approximation algorithm” we propose an algorithm to approximate the likelihoods and their derivatives by dynamically truncating the state space and using the fact that (4) can be written as a product of vectors and matrices. Let *ϕ*_*σ*_be the density of the normal distribution with mean zero and standard deviation *σ*. Then

$$\begin{array}{l} \;f\left(O(t_{1}),\ldots ,O(t_{R})|X(t_{1})=x_{1},\ldots ,X(t_{R})=x_{R}\right)\\ =\overset{R}{\underset{\ell =1}{\prod }}\overset{d}{\underset{i=1}{\prod }}f\left(O_{i}(t_{\ell })|X_{i}(t_{\ell })=x_{\mathrm{i\ell}}\right)\\ =\overset{R}{\underset{\ell =1}{\prod }}\overset{d}{\underset{i=1}{\prod }}\phi_{\sigma }(O_{i}(t_{\ell })-x_{\mathrm{i\ell}}), \end{array}$$

where **x**_*ℓ*_=(*x*_1*ℓ*_,…,*x*_*dℓ*_). If we write *w*(**x**_*ℓ*_) for $\overset{d}{\underset{i=1}{\prod }}\phi_{\sigma }(O_{i}(t_{\ell })-x_{\mathrm{i\ell}})$, then the sequence **x**_1_,…,**x**_*R*_ has “weight” $\overset{R}{\underset{\ell =1}{\prod }}w(x_{\ell })$ and, thus,

(7) $$ℒ=\underset{x_{1}}{\sum }\ldots \underset{x_{R}}{\sum }\mathrm{Pr}\;\left(X(t_{1})=x_{1},\ldots ,X(t_{R})=x_{R}\right)\overset{R}{\underset{\ell =1}{\prod }}w(x_{\ell }).$$

Moreover, for the probability of the sequence **x**_1_,…,**x**_*R*_we have

$$\begin{array}{lll} & \;\mathrm{Pr}\left(X(t_{1})=x_{1},\ldots ,X(t_{R})=x_{R}\right)=p(x_{1},t_{1})P_{2}(x_{1},x_{2})\ldots \; & \;\\ & \;P_{R}(x_{R-1},x_{R})\; & \; \end{array}$$

where *P*_*ℓ*_(**x**,**y**)=Pr(**X**(*t*_*ℓ*_)=**y**∣**X**(*t*_*ℓ*−1_)=**x**) for *d*-dimensional population vectors **x**and **y**. Hence, (7) can be written as

(8) $$\begin{array}{l} ℒ=\underset{x_{1}}{\sum }p(x_{1},t_{1})w(x_{1})\underset{x_{2}}{\sum }P_{2}(x_{1},x_{2})w(x_{2})\ldots \\ \;\underset{x_{R}}{\sum }P_{R}(x_{R-1},x_{R})w(x_{R}). \end{array}$$

Assume that *d*=*n* and let *P*_*ℓ*_ be the matrix with entries *P*_*ℓ*_(**x**,**y**) for all possible states **x**,**y**. Note that *P*_*ℓ*_ is the transition probability matrix of **X**for time step *t*_*ℓ*_−*t*_*ℓ*−1_ and thus the general solution $e^{Q(t_{\ell }-t_{\ell -1})}$ of the Kolmogorov forward and backward differential equations

$$\frac{d}{\mathrm{dt}}P_{\ell }=QP_{\ell },\;\frac{d}{\mathrm{dt}}P_{\ell }=P_{\ell }\mathrm{Q.}$$

In this case, using **p**(*t*_1_)=**p**(*t*_0_)*P*_1_with *t*_0_=0, we can write (8) in matrix-vector form as

(9) $$ℒ=p(t_{0})P_{1}W_{1}P_{2}W_{2}\ldots P_{R}W_{R}e.$$

Here, **e** is the vector with all entries equal to one and *W*_*ℓ*_ is a diagonal matrix whose diagonal entries are all equal to *w*(**x**_*ℓ*_) with *ℓ* ∈ {1,…,*R*}, where *W*_*ℓ*_ is of the same size as *P*_*ℓ*_.

If *d* <*n*, then we still have the same matrix-vector product as in (9), but define the weight *w*(**x**) of an *n*-dimensional population vector as

$$w(x_{1},\ldots ,x_{n})=\overset{d}{\underset{i=1}{\prod }}\phi_{\sigma }(O_{i}(t_{\ell })-x_{i}),$$

i.e. the populations of the unobserved species have no influence on the weight.

Since it is in general not possible to analytically obtain parameters that maximize  ℒ, we use numerical optimization techniques to find **c**^∗^, **x**(0)^∗^ and *σ*^∗^. Typically, such techniques iterate over values of **c**, **x**(0) and *σ* and increase the likelihood ℒ(c,σ) by following the gradient. Therefore, we need to calculate the derivatives $\frac{\partial }{\partial c_{j}}ℒ$, $\frac{\partial }{\partial x_{i}(0)}ℒ$ and $\frac{\partial }{\partial \sigma }ℒ$. For $\frac{\partial }{\partial c_{j}}ℒ$ we obtain

(10) $$\begin{array}{c} \frac{\partial }{\partial c_{j}}ℒ=\frac{\partial }{\partial c_{j}}\left(p(t_{0})P_{1}W_{1}P_{2}W_{2}\ldots P_{R}W_{R}e\right)\\ \;=p(t_{0})\left(\overset{R}{\underset{\ell =1}{\sum }}\left(\frac{\partial }{\partial c_{j}}P_{\ell }\right)W_{\ell }\underset{\ell^{\prime }\neq \ell }{\prod }P_{\ell^{\prime }}W_{\ell^{\prime }}\right)e. \end{array}$$

The derivative of  ℒ w.r.t. *x*_*i*_(0) and *σ* is derived analogously. The only difference is that **p**(*t*_0_) is dependent on *x*_*i*_(0) and *P*_1_,…,*P*_*R*_ are independent of *σ*but *W*_1_,…,*W*_*R*_ depend on *σ*. It is also important to note that expressions for partial derivatives of second order can be derived in a similar way. These derivatives can then be used for an efficient gradient-based local optimization.

For *K* > 1 observation sequences we can maximize the log-likelihood

(11) $$\log\overset{K}{\underset{k=1}{\prod }}{ℒ}_{k}=\overset{K}{\underset{k=1}{\sum }}\log{ℒ}_{k},$$

instead of the likelihood in (6). Note that the derivatives are then given by

(12) $$\frac{\partial }{\partial \lambda }\overset{K}{\underset{k=1}{\sum }}\log{ℒ}_{k}=\overset{K}{\underset{k=1}{\sum }}\frac{\frac{\partial }{\partial \lambda }{ℒ}_{k}}{{ℒ}_{k}},$$

where *λ* is *c*_*j*_, *x*_*i*_(0) or *σ*. It is also important to note that only the weights *w*(**x**_*ℓ*_) depend on *k*, that is, on the observed sequence **O**^*k*^(*t*_1_),…,**O**^*k*^(*t*_*R*_). Thus, when we compute ${ℒ}_{k}$ based on (9) we use for all *k* the same transition matrices *P*_1_,…,*P*_*R*_and the same initial conditions **p**(*t*_0_), but possibly different matrices *W*_1_,…,*W*_*R*_.

## Numerical approximation algorithm

In this section, we focus on the numerical approximation of the likelihood and the corresponding derivatives. Our algorithm calculates an approximation of the likelihood based on (9) by traversing the matrix-vector product from the left to the right. The main idea behind the algorithm is that instead of explicitly computing the matrices *P*_*ℓ*_, we express the vector-matrix product **u**(*t*_*ℓ*−1_)*P*_*ℓ*_as a system of ODEs similar to the CME (cf. Equation (1)). Note that even though *P*_*ℓ*_is sparse the number of states may be very large or infinite, in which case we cannot compute *P*_*ℓ*_ explicitly. Let **u**(*t*_0_),…,**u**(*t*_*R*_) be row vectors that are obtained during the iteration over time points *t*_0_,…,*t*_*R*_, that is, we define  ℒ recursively as $ℒ=u(t_{R})e$ with **u**(*t*_0_)=**p**(*t*_0_) and

$$u(t_{\ell })=u(t_{\ell -1})P_{\ell }W_{\ell }\;\text{for all}\;1\leq \ell \leq R,$$

where *t*_0_=0. We solve *R* systems of ODEs

(13) $$\frac{d}{\mathrm{dt}}\tilde{u}(t)=\tilde{u}(t)Q$$

with initial condition $\tilde{u}(t_{\ell -1})=u(t_{\ell -1})$ for the time interval *t*_*ℓ*−1_*t*_*ℓ*_) where *ℓ* ∈ {1,…,*R*}. After solving the *ℓ*-th system of ODEs we set $u(t_{\ell })=\tilde{u}(t_{\ell })W_{\ell }$ and finally compute $ℒ=u(t_{R})e$. We remark that this is the same as solving the CME for different initial conditions and due to the largeness problem of the state space we use the dynamic truncation of the state space that we proposed in previous work [17]. The idea is to consider only the most relevant equations of the system (13), i.e., the equations that correspond to those states **x** where the relative contribution $ũ(x,t)/(\tilde{u}(t_{\ell })e)$ is greater than a threshold *δ*. Since during the integration the contribution of a state might increase or decrease we add/remove equations on-the-fly depending on the current contribution of the corresponding state. Note that the structure of the CME allows us to determine in a simple way which states will become relevant in the next integration step. For a small time step of length *h* we know that the probability being moved from state **x**−**v**_*j*_ to **x** is approximately *α*_*j*_(**x**−**v**_*j*_)*h*. Thus, we can simply check whether a state that receives a certain probability inflow receives more than the threshold. In this case we consider the corresponding equation in (13). Otherwise, if a state does not receive enough probability inflow, we do not consider it in (13). For more details on this technique we refer to [17].

Since the vectors $\tilde{u}(t_{\ell })$ do not sum up to one, we scale all entries by multiplication with $1/(\tilde{u}(t_{\ell })e)$. This simplifies the truncation of the state space using the significance threshold *δ* since after scaling it can be interpreted as a probability. In order to obtain the correct (unscaled) likelihood, we compute  ℒ as $ℒ=\overset{R}{\underset{\ell =1}{\prod }}\tilde{u}(t_{\ell })e$. For our numerical implementation we used a threshold of *δ*=10^−15^ and handle the derivatives of  ℒ in a similar way. To shorten our presentation, we only consider the derivative $\frac{\partial }{\partial c_{j}}ℒ$ in the sequel of the article. Iterative schemes for $\frac{\partial }{\partial \sigma }ℒ$ and $\frac{\partial }{\partial x_{i}(0)}ℒ$ are derived analogously. From (10) we obtain $\frac{\partial }{\partial c_{j}}ℒ=u_{j}(t_{R})e$ with **u**_*j*_(*t*_0_)=**0** and

$$\;u_{j}(t_{\ell })=(u_{j}(t_{\ell -1})P_{\ell }+u(t_{\ell -1})\frac{\partial }{\partial c_{j}}P_{\ell })W_{\ell }\;\text{for all}\;1\leq \ell \leq R,$$

where **0** is the vector with all entries zero. Thus, during the solution of the *ℓ*-th ODE in (13) we simultaneously solve

(14) $$\frac{d}{\mathrm{dt}}{\tilde{u}}_{j}(t)={\tilde{u}}_{j}(t)Q+\tilde{u}(t)\frac{\partial }{\partial c_{j}}Q$$

with initial condition ${\tilde{u}}_{j}(t_{\ell -1})=u_{j}(t_{\ell -1})$ for the time interval [*t*_*ℓ*−1_,*t*_*ℓ*_). As above, we set $u_{j}(t_{\ell })={\tilde{u}}_{j}(t_{\ell })W_{\ell }$ and obtain $\frac{\partial }{\partial c_{j}}ℒ$ as **u**_*j*_(*t*_*R*_)**e**.

Solving (13) and (14) simultaneously is equivalent to the computation of the partial derivatives in (2) with different initial conditions. Numerical experiments show that the approximation errors of the likelihood and its derivatives are of the same order of magnitude as those of the transient probabilities and their derivatives. For instance, for a finite-state enzymatic reaction system that is small enough to be solved without truncation we found that the maximum absolute error in the approximations of the vectors **p**(*t*) and **s**_*λ*_(*t*) is 10^−8^ if the truncation threshold is *δ*=10^−15^(details not shown).

In the case of *K* observation sequences we repeat the above algorithm in order to sequentially compute ${ℒ}_{k}$ for *k* ∈ {1,…,*K*}. We exploit (11) and (12) to compute the total log-likelihood and its derivatives as a sum of individual terms. In a similar way, second derivatives can be approximated. Obviously, it is possible to parallelize the algorithm by computing ${ℒ}_{k}$ in parallel for all *k*.

In order to find values for which the likelihood becomes maximal, global optimization techniques can be applied. Those techniques usually use a heuristic for different initial values of the parameters and then follow the gradient to find local optima of the likelihood. In this step the algorithm proposed above is used since it approximates the gradient of the likelihood. The approximated global optimum is then chosen as the minimum/maximum of the local optima, i.e, we determine those values of the parameters that give the largest likelihood. Clearly, this is an approximation and we cannot guarantee that the global optimum was found. Note that this would also be the case if we could compute the exact likelihood. If, however, a good heuristic for the starting points is chosen and the number of starting points is large, then it is likely that the approximation is accurate. Moreover, since we have approximated the second derivative of the log-likelihood, we can compute the entries of the Fisher information matrix and use this to approximate the standard deviation of the estimated parameters, i.e., we consider the square root of the diagonal entries of the inverse of a matrix *H* which is the Hessian matrix of the negative log-likelihood. Assuming that the second derivative of the log-likelihood is computed exactly, these entries asymptotically tend to the standard deviations of the estimated parameters.

We remark that the approximation proposed above becomes unfeasible if the reaction network contains species with high molecule numbers since in this case the number of states that have to be considered is very large. A numerical approximation of the likelihood is, as the solution of the CME, only possible if the expected populations of all species remain small (at most a few hundreds) and if the dimension of the process is not too large. Moreover, if many parameters have to be estimated, the search space of the optimization problem may become unfeasibly large. It is however straightforward to parallelize local optimizations starting from different initial point.

## Numerical results

In this section we present numerical results of our parameter estimation algorithm applied to two models, the simple gene expression in Example 1 and a multi-attractor model. The corresponding SBML files are provided as Additional files 1 and 2. For both models, we generated time series data using Monte-Carlo simulation where we added white noise to represent measurement errors, i.e. we added random terms to the populations that follow a normal distribution with mean zero and a standard deviation of *σ*. Our algorithm for the approximation of the likelihood is implemented in C++ and linked to MATLAB’s optimization toolbox [20] which we use to minimize the negative log-likelihood. The global optimization method (Matlab’s GlobalSearch [21]) uses a scatter-search algorithm to generate a set of trial points (potential starting points) and heuristically decides when to perform a local optimization. We ran our experiments on an Intel Core i7 at 2.8 GHz with 8 GB main memory.

### Simple gene expression

**Figure 1:**
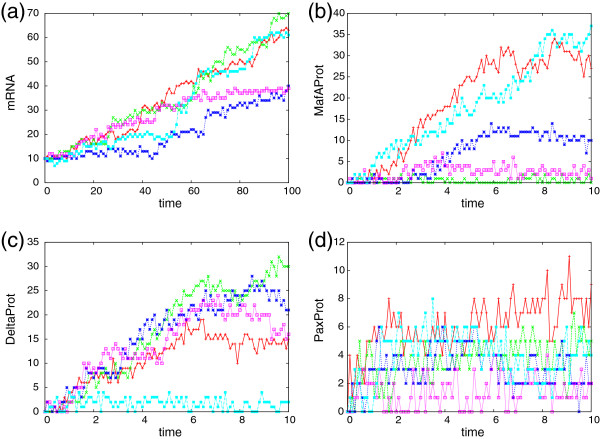
**Time series data.** Generated observation sequences for the gene expression **(a)** and multi-attractor **(b)**–**(d)** models. Each plot shows *K*=5 sequences with *R*=100 time points.

**Figure 2:**
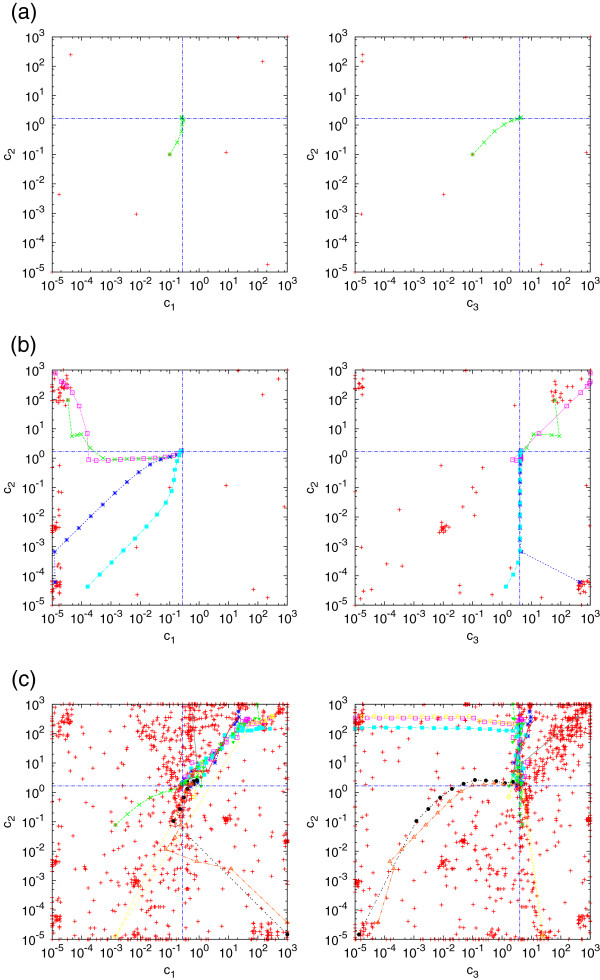
**Start points and gradient convergence of the optimization procedure for the gene expression example: Red pluses show the potential start points.** We use 10,100, and 1000 start points in case **(a)**, **(b)**, and **(c)**, respectively. The markers that are connected by lines show the iterative steps of the gradient convergence while the dashed blue line shows the true values of the parameters. We chose *K*=5,*R*=100 and assume that the parameters are in the range [10^−5^,10^3^].

For our first model, the simple gene expression as introduced in Example 1, we chose the same parameters as Reinker et al. [4] multiplied by a factor of 10, i.e., **c**=(0.270,1.667,4.0) and as the initial condition we have ten mRNA molecules and the DNA is inactive. We generated *K* observation sequences of length *T*=100.0 and observed all species at *R* equidistant observation time points. We added white noise with standard deviation *σ*=1.0 to the observed mRNA molecule numbers at each observation time point. For the case *K*=5,*R*=100 we plot the generated observation sequences in Figure 1. We estimated the reaction rate constants, the initial molecule numbers, and the parameter *σ* of the measurement errors for the case *K*=5,*R*=100 where we chose the interval [10^−5^,10^3^ as a constraint for the rate constants, the interval [0,100] for the initial number of mRNA molecules and [0,5] for *σ*. Since we use a global optimization method, the running time of our method depends on the number of trial points generated by GlobalSearch. In Figure 2 we plot the trial points (red points) and local optimization runs (differently colored lines) for the case of 10 (a), 100 (b) and 1000 (c) trial points. The intersection of the dashed blue lines represents the location of the original parameters. In the case of ten trial points, the running time was about one minute and the local optimization was performed only once. In the case of 100 and 1000 trial points, the running times were about 22 min and 1.9 h, respectively and several local optimization runs converged in nearly the same point. However, we remark that in general the landscape of the target function might have multiple local minima and require more trial points resulting in longer running times.

We ran experiments for varying values of *K* and *R* (*K*,*R* ∈ {1,2,5,10,20,50,100}) to get insights whether for this network it is more advantageous to have many observation sequences with long observation intervals or few observation sequences with a short time between two successive observations. In addition, we ran the same experiments with the restriction that only the number of mRNA molecules was observable but not the state of the gene. In both cases we approximated the standard deviations of our estimators as a measure of quality by repeating our estimation procedure 100 times and by the Fisher information matrix as explained at the end of the previous section. We used 100 trial points for the global optimization procedure and chose tighter constraints than above for the rate constants ([0.01,1] for *c*_1_ and [0.1,10] for *c*_2_,*c*_3_) to have a convenient total running time.

**Figure 3:**
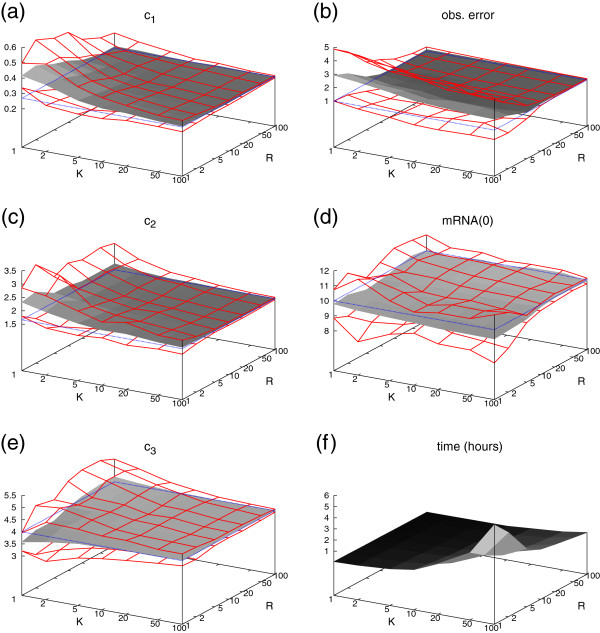
**Results of the gene expression case study with observable gene state.** The dotted blue rectangle gives the true value of *c*_1_, *c*_2_, *c*_3_, *σ*(obs. error), and mRNA(0). The red grid corresponds to the approximated standard deviation of the estimators.

**Figure 4:**
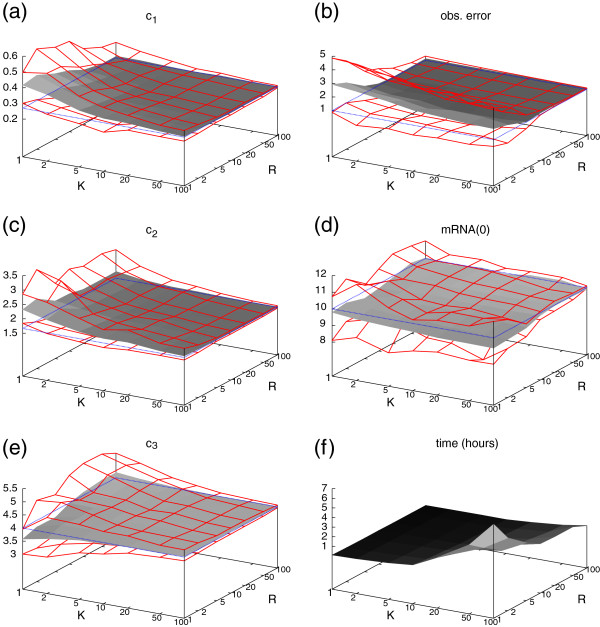
Results of the gene expression case study (as in Figure 3) but the state of the gene is not observed.

The results are depicted in Figure 3 for the fully observable system and in Figure 4 for the restricted system, where the state of the gene was not visible. In these figures we present the estimations of the parameters *c*_1_, *c*_2_, *c*_3_, *σ*, and an estimation of the initial condition, i.e. the number of mRNA molecules at time point *t*=0. Moreover, we give the total running time of the procedure (Figures 3f and 4f). Our results are plotted as a gray landscape for all combinations of *K* and *R*. The estimates are bounded by a red grid enclosing an environment of one standard deviation around the respective average over all 100 estimates that we approximated. The real value of the parameter is indicated by a dotted blue rectangle.

At first, we remark that neither the quality of the estimation nor the running time of our algorithm is significantly dependent on whether we observe the state of the gene in addition to the mRNA level or not. Moreover, concerning the estimation of all of the parameters, one can witness that the estimates converge more quickly against the real values along the *K* axis than the *R* axis and also the standard deviations decrease faster. Consequently, at least for the gene expression model, it is more advantageous to increase the number of observation sequences, than the number of measurements per sequence. For example, *K*=100 sequences with only one observation each already provide enough information to estimate *c*_1_ up to a relative error of around 2.1%. Unfortunately, in this case the computation time is the highest since we have to compute *K* individual likelihoods (one for each observation sequence). Moreover, if *R* is small then the truncation of the state space is less efficient. The reason is that we have to integrate for a long time until we multiply with the weight matrix *W*_*ℓ*_. After this multiplication we decide which states contribute significantly to the likelihood and which states are neglected. We can, however, trade off accuracy against running time by varying *K*.

For the measurement noise parameter *σ*we see that it is more advantageous to increase *R*. Even five observation sequences with a high number of observations per sequence (*R*=100) suffice to estimate the noise up to a relative error of around 10.2%. For the estimation of the initial conditions, both *K* and *R* seem to play an equally important role.

The standard deviations of the estimators give information about the accuracy of the estimation. In order to approximate the standard deviation we used statistics over 100 repeated experiments. In a realistic setting one would rather use the Fisher information matrix to approximate the standard deviation of the estimators since it is in most cases difficult to observe 100·*K*observation sequences of a real system. Therefore we compare the results of one experiment with *K* observation sequences and standard deviations approximated using the Fisher information matrix to the case where the experiment is repeated 100 times. The results for varying values of *K* and *R* are given in Table 1 We observe that the approximation using the Fisher information matrix is in most cases close to the approximation based on 100 repetitions as long as *K* and *R* are not too small. This comes from the fact that the Fisher information matrix converges to the true standard deviation as the sample size increases.

**Table 1:** Different approximations of the standard deviations of the estimators

**Method**	***K***	***R***	***c*_1_**	***c*_2_**	***c*_3_**	***σ***	**mRNA(0)**
Fisher inf. matrix	10	10	0.0545104	0.561963	0.935324	0.364339	0.639471
100 experiments			0.0358142	0.198700	0.262223	0.392884	0.490305
Fisher inf. matrix	20	20	0.0324508	0.299487	0.451476	0.174095	0.594820
100 experiments			0.0304157	0.167431	0.287471	0.134506	0.436059
Fisher inf. matrix	50	50	0.0139185	0.110709	0.152229	0.0440282	0.238033
100 experiments			0.0140331	0.078516	0.146232	0.0353837	0.183888
Fisher inf. matrix	100	100	0.00866066	0.0548249	0.0728129	0.0182564	0.208469
100 experiments			0.00691956	0.0430123	0.0641821	0.0217544	0.187968

### Multi-attractor model

**Figure 5:**
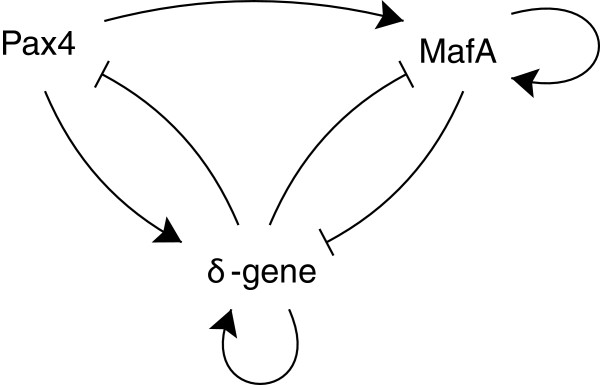
Illustration of the multi-attractor model.

Our final example is a part of the multi-attractor model considered by Zhou et al. [22]. It consists of the three genes *MafA*, *Pax4*, and *δ*-gene, which interact with each other as illustrated in Figure 5. The corresponding proteins bind to specific promoter regions on the DNA and (de-)activate the genes. The reaction network has 2^3^different gene states, also called modes, since each gene can be on or off. It is infinite in three dimensions since for the proteins there is no fixed upper bound. The edges between the nodes in Figure 5 show whether the protein of a specific gene can bind to the promoter region of another gene. Moreover, edges with normal arrow heads correspond to binding without inhibition while the edges with line heads show inhibition.

**Figure 6:**
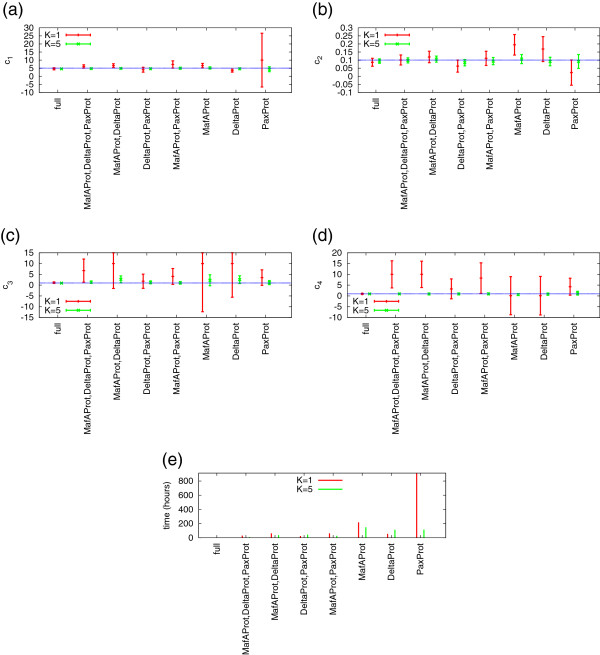
**Parameter estimation results for the multi-attractor model.** The *x*-axis shows the species that were observed during the estimation procedure. The dotted blue line corresponds to the true value of *c*_1_, *c*_2_, *c*_3_, and *c*_4_, respectively. The error bars in **(a)**–**(d)** show the mean (plus/minus the standard deviation) of the estimators. In **(e)** we plot the running time of the estimation procedure.

We list all 24 reactions in Table 2 For simplicity we first assume that there is a common rate constant for all protein production reactions (*p*), for all protein degradations (*d*), binding (*b*), and unbinding (*u*) reactions. We further assume that initially all genes are active and no proteins are present. For the rate constants we chose **c**=(*p*,*d*,*b*,*u*)=(5.0,0.1,1.0,1.0) and generated *K* ∈ {1,5} sample paths of length *T*=10.0. We added normally distributed noise with zero mean and standard deviation *σ*=1.0 to the protein levels at each of the *R*=100 observation time points. Plots of the generated observation sequences are presented in Figure 1 b–d for the case *K*=5. For the global optimization we used ten trial points. We chose the interval [0.1,10] as a constraint for the rate constants *p*,*b*,*u* and the interval [0.01,1] for *d*. We estimated the parameters for all 2^3^−1=7 possibilities of observing or not observing the three protein numbers where at least one of them had to be observable. In addition we repeated the parameter estimation for the fully observable system where in addition to the three proteins also the state of the genes was observed. The results are depicted in Figure 6 where the *x*-axis of the plots refers to the observed proteins. For instance, the third entry on the *x*-axis of the plot in Figure 6 a shows the result of the estimation of parameter *c*_1_=5 based on observation sequences where only the molecule numbers of the proteins MafAProt and DeltaProt were observed. For this case study, we used the Fisher information matrix to approximate the standard deviations of our estimators, plotted as bars in Figure 6 with the estimated parameter as midpoint. The fully observable case is labelled by “full”.

**Table 2:** Chemical reactions of the multi-attractor model

PaxDna	$\overset{p}{\to }$	PaxDna + PaxProt
PaxProt	$\overset{d}{\to }$	*∅*
PaxDna + DeltaProt	$\overset{b}{\to }$	PaxDnaDeltaProt
PaxDnaDeltaProt	$\overset{u}{\to }$	PaxDna + DeltaProt
MafADna	$\overset{p}{\to }$	MafADna + MafAProt
MafAProt	$\overset{d}{\to }$	*∅*
MafADna + PaxProt	$\overset{b}{\to }$	MafADnaPaxProt
MafADnaPaxProt	$\overset{u}{\to }$	MafADna + PaxProt
MafADnaPaxProt	$\overset{p}{\to }$	MafADnaPaxProt + MafAProt
MafADna + MafAProt	$\overset{b}{\to }$	MafADnaMafAProt
MafADnaMafAProt	$\overset{u}{\to }$	MafADna + MafAProt
MafADnaMafAProt	$\overset{p}{\to }$	MafADnaMafAProt + MafAProt
MafADna + DeltaProt	$\overset{b}{\to }$	MafADnaDeltaProt
MafADnaDeltaProt	$\overset{u}{\to }$	MafADna + DeltaProt
DeltaDna	$\overset{p}{\to }$	DeltaDna + DeltaProt
DeltaProt	$\overset{d}{\to }$	*∅*
DeltaDna + PaxProt	$\overset{b}{\to }$	DeltaDnaPaxProt
DeltaDnaPaxProt	$\overset{u}{\to }$	DeltaDna + PaxProt
DeltaDnaPaxProt	$\overset{p}{\to }$	DeltaDnaPaxProt + DeltaProt
DeltaDna + MafAProt	$\overset{b}{\to }$	DeltaDnaMafAProt
DeltaDnaMafAProt	$\overset{u}{\to }$	DeltaDna + MafAProt
DeltaDna + DeltaProt	$\overset{b}{\to }$	DeltaDnaDeltaProt
DeltaDnaDeltaProt	$\overset{u}{\to }$	DeltaDna + DeltaProt
DeltaDnaDeltaProt	$\overset{p}{\to }$	DeltaDnaDeltaProt + DeltaProt

We observe in Figure 6 that as expected the accuracy of the estimation and the running time of our algorithm is best when we have full observability of the system and gets worse with an increasing number of unobservable species. Still the estimation quality is very high when five observation sequences are provided for almost all combinations and parameters. When only one observation sequence is given (*K*=1), the parameter estimation becomes unreliable and time consuming. This comes from the fact that the quality of the approximation highly depends on the generated observation sequence. It is possible to get much better and faster approximations with a single observation sequence. However, we did not optimize our results but generated one random observation sequence and ran our estimation procedure once based on this.

**Figure 7:**
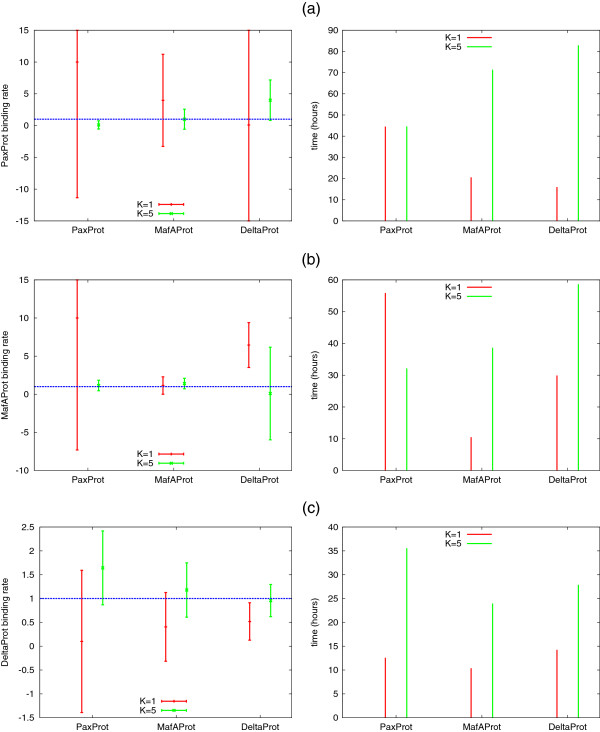
Results of the multi-attractor (as in Figure 6), but we estimate the binding rate of each protein independently.

Recall that we chose common parameters *p*,*d*,*b*,*u*for production, degradation, and (un-)binding for all three protein species. Next we “decouple” the binding rates and estimate the binding rate of each protein independently. We illustrate our results in Figure 7. Again, in case of a single observation sequence (*K*=1) the estimation is unreliable in most cases. If the true value of the parameter is unknown, then the high standard deviation shows that more information (more observation sequences) is necessary to estimate the parameter. In order to estimate the binding rate of PaxProt, we see that observing MafAProt yields the best result while for the binding rate of MafAProt observing PaxProt is best. Only for the binding rate of DeltaProt, the best results are obtained when the corresponding protein (DeltaProt) is observed. The running times of the estimation procedure are between 10 and 80 h, usually increase with *K* and depend on the observation sequences.

In Table 3 we list the results of estimating the production rate 5.0 in the multi-attractor model where we chose *R*=100. More precisely, we estimated the production rate of each protein independently when the other two proteins were observed. Since the population of the PaxProt is significantly smaller than the populations of the other two proteins, its production rate is more difficult to estimate. The production rate of MafAProt is accurately estimated even if only a single observation sequence is considered. For estimating the production rate of DeltaProt, *K*=5 observation sequences are necessary to get an accurate result.

**Table 3:** Production rate estimation in the multi-attractor model

**Protein**	***K***	**Estimated rate constant**	**Standard deviation**	**Time (hours)**	**Observed proteins**
PaxProt	1	10.0	13.6159	7.45	MafAProt, DeltaProt
	5	0.5693	2.1842	6.34	
MafAProt	1	4.9998	4.9884	11.62	PaxProt, DeltaProt
	5	5.4853	2.3873	13.86	
DeltaProt	1	2.5453	1.8075	4.35	PaxProt, MafAProt
	5	5.3646	1.4682	12.39	

Finally, we remark that for the multi-attractor model it seems difficult to predict whether for a given parameter the observation of a certain set of proteins yields a good accuracy or not. It can, however, be hypothesized that, if we want to accurately estimate the rate constant of a certain chemical reaction, then we should observe as many of the involved species as possible. Moreover, it is reasonable that constants of reactions that occur less often are more difficult to estimate (such as the production of PaxProt). In such a case more observation sequences are necessary to provide reliable information about the speed of the reaction.

## Conclusion

Parameter inference for stochastic models of cellular processes demands huge computational resources. We proposed an efficient numerical method to approximate maximum likelihood estimators for a given set of observations. We consider the case where the observations are subject to measurement errors and where only the molecule numbers of some of the chemical species are observed at certain points in time. In our experiments we show that if the observations provide sufficient information then parameters can be accurately identified. If only little information is available then the approximations of the standard deviations of the estimators indicate whether more observations are necessary to accurately calibrate certain parameters.

As future work we plan a comparison of our technique to parameter estimation based on Bayesian inference. In addition, we will examine whether a combination of methods based on prior knowledge and the maximum likelihood method is useful. Future plans further include parameter estimation methods for systems where some chemical species have small molecule numbers while others are high rendering a purely discrete representation infeasible. In such cases, hybrid models are advantageous where large populations are represented by continuous deterministic variables while small populations are still described by discrete random variables [23].

## Competing interests

The authors declare that they have no competing interests.

## Supplementary Material

Additional file 1 SBML file of the gene expression example.
• File name: genexpression.xml
• File format: SBML (see http://www.sbml.org/sbml/level2/version4)
• File extension: xml Click here for file

Additional file 2 SBML file of the multiattractor model.
• File name: multiattractor.xml
• File format: SBML (see http://www.sbml.org/sbml/level2/version4)
• File extension: xml Click here for file
